# Identifying co-endemic areas for major filarial infections in sub-Saharan Africa: seeking synergies and preventing severe adverse events during mass drug administration campaigns

**DOI:** 10.1186/s13071-018-2655-5

**Published:** 2018-01-31

**Authors:** Jorge Cano, Maria-Gloria Basáñez, Simon J. O’Hanlon, Afework H. Tekle, Samuel Wanji, Honorat G. Zouré, Maria P. Rebollo, Rachel L. Pullan

**Affiliations:** 1 Department of Disease Control, Faculty of Infectious & Tropical Diseases, London School of Hygiene & Tropical Medicine, London, UK; 2 London Centre for Neglected Tropical Disease Research, Department of Infectious Disease Epidemiology, School of Public Health, Faculty of Medicine (St Mary’s campus), Imperial College London, London, UK; 3 Department of Biochemistry and Microbiology, University of Buea, P.O. Box 63, Buea, Cameroon; 4 Research Foundation in Tropical Medicine and the Environment, Buea, Cameroon; 5 Former African Programme for Onchocerciasis Control Programme, Ouagadougou, Burkina Faso; 6 Expanded Special Programme for Elimination of Neglected Tropical Diseases (ESPEN), Brazzaville, Republic of Congo

**Keywords:** Filariasis, Onchocerciasis, Lymphatic filariasis, Loiasis, Severe adverse events, Ivermectin, DEC, Albendazole, Mapping, GIS

## Abstract

**Background:**

Onchocerciasis and lymphatic filariasis (LF) are major filarial infections targeted for elimination in most endemic sub-Saharan Africa (SSA) countries by 2020/2025. The current control strategies are built upon community-directed mass administration of ivermectin (CDTI) for onchocerciasis, and ivermectin plus albendazole for LF, with evidence pointing towards the potential for novel drug regimens. When distributing microfilaricides however, considerable care is needed to minimise the risk of severe adverse events (SAEs) in areas that are co-endemic for onchocerciasis or LF and loiasis. This work aims to combine previously published predictive risk maps for onchocerciasis, LF and loiasis to (i) explore the scale of spatial heterogeneity in co-distributions, (ii) delineate target populations for different treatment strategies, and (iii) quantify populations at risk of SAEs across the continent.

**Methods:**

Geographical co-endemicity of filarial infections prior to the implementation of large-scale mass treatment interventions was analysed by combining a contemporary LF endemicity map with predictive prevalence maps of onchocerciasis and loiasis. Potential treatment strategies were geographically delineated according to the level of co-endemicity and estimated transmission intensity.

**Results:**

In total, an estimated 251 million people live in areas of LF and/or onchocerciasis transmission in SSA, based on 2015 population estimates. Of these, 96 million live in areas co-endemic for both LF and onchocerciasis, providing opportunities for integrated control programmes, and 83 million live in LF-monoendemic areas potentially targetable for the novel ivermectin-diethylcarbamazine-albendazole (IDA) triple therapy. Only 4% of the at-risk population live in areas co-endemic with high loiasis transmission, representing up to 1.2 million individuals at high risk of experiencing SAEs if treated with ivermectin. In these areas, alternative treatment strategies should be explored, including biannual albendazole monotherapy for LF (1.4 million individuals) and ‘test-and-treat’ strategies (8.7 million individuals) for onchocerciasis.

**Conclusions:**

These maps are intended to initiate discussion around the potential for tailored treatment strategies, and highlight populations at risk of SAEs. Further work is required to test and refine strategies in programmatic settings, providing the empirical evidence needed to guide efforts towards the 2020/2025 goals and beyond.

**Electronic supplementary material:**

The online version of this article (10.1186/s13071-018-2655-5) contains supplementary material, which is available to authorized users.

## Background

There are at least three filarial nematode diseases of public health importance in sub-Saharan Africa (SSA), namely, lymphatic filariasis (LF; caused in SSA by *Wuchereria bancrofti*), onchocerciasis (caused by *Onchocerca volvulus*), and loiasis (caused by *Loa loa*). Infection with these parasites is responsible for significant morbidity across the continent, causing elephantiasis, river blindness, and eye worm, respectively [1–3]. Whilst loiasis is not yet included within the World Health Organization’s (WHO) list of neglected tropical diseases (NTDs), LF and onchocerciasis are targeted by the WHO 2012 Roadmap on NTDs [4] for elimination in selected African countries by 2020 using preventive chemotherapy. This strategy is implemented through community-wide mass drug administration (MDA), delivered yearly (and in some cases twice yearly) to all at-risk populations until transmission has been interrupted, combined with vector control measures where feasible [4].

Large-scale MDA programmes, implemented locally in endemic communities but coordinated and supported regionally, have been ongoing in Africa for over 25 years, first for onchocerciasis [5] and since 2000 for LF [6]. These are widely considered among the most successful and cost-effective public health interventions ever launched [5, 7]. Nevertheless, there are important factors limiting their sustainability, including the availability of effective drug regimens that ensure a rapid interruption of transmission. For onchocerciasis, ivermectin has been the only drug used for MDA since Merck & Co. Inc. first announced its donation to endemic countries in 1987 [8], whilst for LF the mainstay treatment is a combination of either diethylcarbamazine (DEC, donated by Eisai Co. Ltd) - in non-onchocerciasis endemic areas - or ivermectin, given annually, plus albendazole (donated by GlaxoSmithKline) [9]. Although relatively safe and efficacious against microfilariae (mf, the larval progeny stage), these regimens are not considered to exert a powerful macrofilaricidal (adult stage killing) effect on the long-lived adult worms. Instead, ivermectin has a temporary sterilising effect on female *O. volvulus* [10] and, in combination with albendazole, also on *W. bancrofti* [11]). Thus in order to interrupt transmission, MDA must be continued, at high levels of treatment coverage and adherence [12], for at least as long as the duration of the reproductive lifespan of the adult worms (ranging from 4 to 12 years for *W. bancrofti* [13] and from 9 to 11 years for *O. volvulus*, with 95% of the worms ending reproduction by the age of 13 to 15 years [14].

For filarial control programmes to be successful in shorter timeframes, regimens that kill or irreversibly sterilise adult worms are required [15]. As an alternative to developing a new compound, it has been suggested that simultaneous provision of triple drug therapy (IDA; ivermectin + DEC + albendazole) may improve LF microfilarial clearance and further impact upon adult worms [16]. A pilot study conducted in Papua New Guinea has reported that single dose IDA treatment rapidly eliminated all *W. bancrofti* mf from peripheral blood. Encouragingly, all participants treated with this regimen remained amicrofilaraemic for at least 2 years following treatment, suggesting sterilisation or killing of adult worms [16]. Recent simulation modelling based on these findings has further suggested that the triple-drug regimen has potential to accelerate the elimination of LF, conditional on achieving high population coverage and low systematic non-adherence to MDA [17]. To appreciate fully the potential of IDA for reducing the duration of MDA interventions against LF, it is imperative that these findings be replicated within larger trial settings.

Whilst IDA may help to accelerate the elimination of LF, it is important to delineate the settings where its use for MDA would be safe and appropriate [18]. One major concern is the risk of severe adverse events (SAEs), which can arise following microfilaricidal medication. In the limited setting of the IDA pilot trial, adverse events were more common in those treated with the triple therapy, although no SAEs were recorded [16]. This may have important implications for programme safety and compliance. DEC cannot be used in areas where onchocerciasis is present, because it induces a strong local inflammation in patients with ocular (*O. volvulus*) mf [19]. Similarly, providing ivermectin or DEC to those with high *Loa loa* microfilarial loads has been associated with SAEs, including neurological sequelae and fatal encephalopathy [20, 21], precluding their use in forest areas throughout much of central Africa [22]. This has led to the recommendation that twice-yearly albendazole be implemented together with distribution of long-lasting insecticidal nets for control of LF in *L. loa* co-endemic areas [23]. Another aspect that may hamper the large-scale implementation of IDA in only-LF endemic areas is the risk for individuals migrating from onchocerciasis endemic areas, a frequent occurrence in central African countries. This risk could be mitigated somewhat by obtaining information about the history of residence of those individuals to be treated.

Unfortunately, albendazole alone does not kill *O. volvulus* macro- or microfilariae [24], reducing the number of potential strategies for the control and elimination of onchocerciasis in *L. loa* co-endemic areas. This is particularly true in areas hypoendemic for *O. volvulus*, where the risk of SAEs in individuals with loiasis outweighs the benefits of deploying ivermectin MDA. Current guidelines, developed by the Mectizan® Expert Committee and the Technical Consultative Committee (MEC/TCC) of the African Programme for Onchocerciasis Control (APOC) recommend to test for *L. loa* infection and treat accordingly (‘test-and-treat’ protocols) when areas to be treated with ivermectin are suspected, or known to be endemic for loiasis [25]. By this approach, the relatively small proportion of *L. loa-*infected individuals at risk of SAEs (those with > 30,000 mf/ml) are identified and excluded from treatment with ivermectin [20, 26]. This can be difficult to implement in practice as the current gold standard for *L. loa* diagnosis (thick-smear microscopy) requires trained personnel in a central laboratory, and so results are not immediately available for decision-making.

These challenges have prompted two areas of research that together comprise an enhanced ‘test and treat’ strategy: novel diagnostics to enable rapid identification of those with high levels of *L. loa* infection in the field in real time [27–29]; and new filaricides to treat onchocerciasis without affecting *L. loa* [30, 31]*.* If ongoing development and field-testing are successful, *L. loa-O. volvulus* co-infected individuals at risk of SAEs could be excluded during ivermectin mass treatment campaigns, and instead treated with an alternative filariacide such as doxycycline [32]. Additionally, a new strategy based on fine scale-mapping of loiasis in onchocerciasis co-endemic areas has also been suggested to improve targeting, on the basis that environmental changes and population movements may have changed the epidemiological scenario depicted by previous RAPLOA surveys [33].

To facilitate adoption of these innovative MDA drug regimens, whilst ensuring the risk of SAEs is minimised, we must pay careful consideration to the co-distribution of these three filarial species. Large-scale surveys for each species have been conducted across most endemic areas in Africa, and geostatistical approaches have been used to predict the geographical distribution and endemicity levels prior to control [34–36]. The extent of spatial heterogeneity in co-distributions across the SSA region however is less clearly defined. Building upon previous work by Kelly-Hope et al. [37, 38], we present an initiative to use available, single-species spatial predictions to delineate co-distribution of these major filarial infections across SSA, enabling enumeration of target populations for different treatment schemes and quantification of populations potentially at-risk of SAEs.

## Methods

### Developing filariases co-endemicity maps for sub-Saharan Africa

The mapping sources used to identify co-endemic filarial infection settings across the continent include contemporary maps of LF endemicity published by the WHO’s Expanded Special Project for Elimination of Neglected Tropical Diseases (ESPEN) and published predictive risk maps for onchocerciasis [34, 39] and loiasis [36] produced by the African Programme for Onchocerciasis Control (APOC). The latter are available together with other epidemiological resources at www.ntdmap.org [40]. Several pragmatic adjustments were made to better adapt these pre-control predictive maps to the contemporary situation, as outlined below. In brief:

(i) The current-day distribution of LF endemicity was obtained from the new NTD portal developed by ESPEN [41] and from the Preventive Chemotherapy and Transmission Control (PCT) databank [42]. According to WHO guidelines, programmatic implementation units (IUs; typically correspond to administrative areas such as districts) are declared as endemic for LF when at least 1 adult (≥ 15-yr) in 100 surveyed has a positive circulating filarial antigen (CFA) test or presents *W. bancrofti* mf in peripheral blood [43]. For areas where endemicity status was unavailable, we used a risk map of predicted LF antigenaemia prevalence developed using geostatistical modelling approaches [35]. Further details are given in Additional file 1: Text S1 and Figure S1.
(ii) For onchocerciasis, we combined two published sources to generate an SSA-wide map. We used gridded maps of predicted prevalence at 5 × 5 km resolution continuous risk surface because, unlike LF, MDA is not always delivered to an entire implementation unit; instead, the eligible population only includes residents of communities considered at risk, namely living in transmission zones [44, 45] within the implementation unit. The first source considered was a map of the estimated prevalence of palpable nodules (onchocercomata) prior to the implementation of control interventions, developed for the region covered by APOC. With the exception of foci where onchocerciasis has been deemed eliminated (see below) all areas with nodule prevalence > 5% (prior to the initiation of control activities) were considered to remain endemic and targetable for control, based on prevalence contour maps and likelihood of sustained local transmission [34, 44]. For West Africa, the region covered by the Onchocerciasis Control Programme in West Africa (OCP), endemicity was classified on the basis of a predictive map of microfilarial prevalence [39]. The onchocerciasis foci considered by the WHO to have been eliminated in certain foci in Mali, Senegal (including the Gambia River Basin, Faleme River Basin and Bakoye River Basin) [46, 47], Nigeria [48], Sudan and Uganda [49–51] and were masked out, although elsewhere transmission still persists [52–56].
(iii) A gridded map of the estimated prevalence of eye worm history (EWH), obtained by interpolating rapid assessment procedure for *Loa loa* (RAPLOA) survey data conducted in 11 loiasis endemic countries, was used as an approximation to loiasis prevalence as described in detail elsewhere [36]. The resulting map was stratified into three areas based on the empirical relationship between prevalence of EWH and high *L. loa* microfilarial loads (≥ 30,000 mf/ml; i.e. the threshold above which ivermectin-induced SAEs may be expected [26]), namely, ≥ 40% EWH prevalence (high risk of SAEs), 20–40% EWH prevalence (lower SAE risk, but enhanced post-treatment monitoring required) and < 20% EWH prevalence (negligible risk of SAEs) [26].

Filarial co-endemicity was explored at IU level, the subnational administrative level considered for MDA interventions. A harmonized IU-level cartography was obtained from Geoconnect (http://www.geoconnect.org/). Overlaid maps were classified according to the co-endemicity classification shown in Table 1 and population estimates for 2015 produced using a gridded population density map for 2015 [57]. Filarial transmission is not usually associated with large urban areas, and so urban areas (defined as areas with population densities ≥ 1000 persons/km^2^) and peri-urban areas (those with > 250 persons/km^2^ within a 15 km distance from urban extension edge) were excluded. Otherwise, our approach assumes that, unless interruption of transmission has been confirmed, the boundaries for transmission remain as they were pre-control, despite reduced prevalence in areas receiving control.

**Table 1:** Potential mass drug administration (or test-and-treat) strategies according to the co-endemicity of filarial infections in Africa

Onchocerciasis	Lymphatic filariasis (LF)	Loiasis, based on EWH^a^			
		Non-endemic	Low (< 20%)	Moderate (20–40%)^b^	High (≥ 40%)
Non-endemic	Endemic	DEC + ALB+IVM	DEC + ALB+IVM	IVM + ALB Enhanced^c^ ATS: T&T	ALB (2/year) + ITN ATS: T&T
	Non-endemic	–	–	–	–
Endemic	Endemic	IVM + ALB	IVM + ALB	IVM + ALB Enhanced ATS: T&T	IVM + ALB +ITN (T&T) Enhanced & MM^d^ ATS: T&T
	Non-endemic	IVM	IVM	IVM Enhanced ATS: T&T	ATS: T&T Enhanced + MM

^a^If assessment of loiasis is based on thick-smear or CellScope Loa, the alternative treatment strategy (ATS) of Test & Treat (T&T) will exclude those with > 30,000 microfilariae/ml and will treat the remainder with ivermectin (IVM). Those excluded from IVM treatment can be offered doxycycline or albendazole twice a year

^b^Re-assessment (by RAPLOA or by parasitological methods) if distance to area with high EWH prevalence is below certain threshold (i.e. 10 km)

^c^The term ‘Enhanced’ refers to post-treatment monitoring of severe adverse events (SAEs). For interruption of transmission, the duration of treatment (e.g. number of rounds) will be determined in part by the level of pre-control LF and/or onchocerciasis endemicity. Treatment coverage (of the total population) should be at least 65% for LF and 80% for onchocerciasis; non-adherence to treatment should be minimised

^d^Enhanced & MM (enhanced surveillance of potential loiasis-related SAEs and medical monitoring at the community for five day safter MDA treatment)

*Abbreviations*: *ALB* albendazole, *ATS* alternative treatment strategy, *DEC* diethylcarbamazine, *EWH* eye worm history, *IVM* ivermectin, *ITN* insecticide-treated nets, *LF* lymphatic filariasis, *MDA* mass drug administration, *RAPLOA* rapid assessment procedure for *Loa loa*, *SAE* severe adverse event, *T&T* Test (for loiasis) and treat those not at risk of SAEs (quantify *L. loa* microfilaraemia and treat those with < 30,000 mf/ml)

All data processing was conducted using ArcGIS 10.3 (ESRI, Redlands, CA, USA) and R v3.3.3 software. Gridded maps of filarial infections were combined to generate an output raster dataset of filarial co-endemicity. Population estimates were extracted by overlaying a gridded map of population density for 2015 [57] with the co-endemicity reclassified map.

## Results and discussion

The resulting maps and figures highlight substantial within-country heterogeneity in the distributions of the three filarial infections, suggesting that to achieve optimal impact safely, tailored treatment strategies need to vary between (and perhaps even within) existing IUs. Suggested treatment strategies for each co-endemicity setting are provided in Table 1.

Aggregating the resulting population estimates (Additional file 1: Table S1) identifies 251 million people living in areas of LF and/or onchocerciasis transmission in SSA (see Additional file 1: Figure S2 for further details of the co-distributions of these two filarial nematode species). Of these, 81 million individuals across the continent live in LF mono-endemic areas and may be eligible for IDA (54% of whom live in eastern Africa), suggesting that this strategy could have a transformative impact across the region. A further 90.1 million live in areas targetable for ivermectin plus albendazole (LF endemic regions without high *L. loa*). Together, these two regimens bring substantial additional benefits due to the wider antiparasitic efficacy of combined albendazole and ivermectin, notably against strongyloidiasis, trichuriasis, enterobiasis and some epidermal parasitic skin diseases, including scabies [58, 59]. Lastly, of these 90.1 million, 79.7 million live in LF-onchocerciasis co-endemic areas, highlighting substantial opportunities for programme integration.

Our estimates clearly differ from figures provided by WHO on people requiring preventive chemotherapy in 2015 (PCT databank, WHO [60]). This may be explained by the alternative source of demographic data used to generate estimates, and by a more precise delineation of onchocerciasis endemic areas based on the geostatistical models. However, it should also be noted that figures for LF endemicity in Middle Africa should be treated with some caution, due to recent observations of cross-reactivity of the immunochromatographic (ICT) test used in LF mapping surveys to *L. loa* infections [61–63]. As a result, some areas currently considered endemic for LF in Middle Africa may require re-evaluation, which may in turn lead to the shrinkage of the LF endemicity map for this region.

**Fig. 1:**
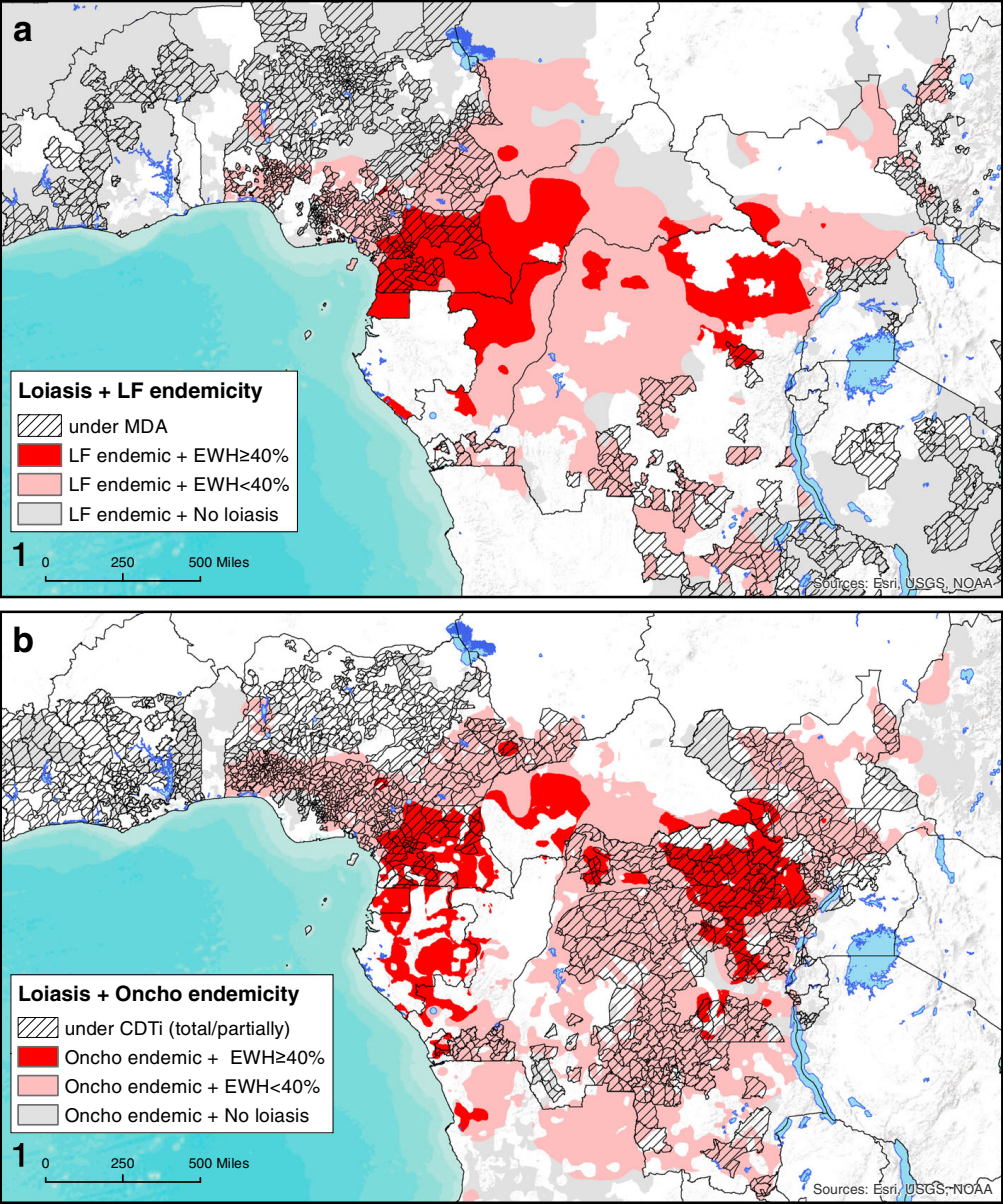
Maps displaying areas currently under MDA treatment (hatched areas) which are co-endemic for loiasis and lymphatic filariasis (**a**) and loiasis and onchocerciasis (**b**). *Abbreviations*: CDTi, community directed treatment with ivermectin; EWH, prevalence of eye worm history; LF, lymphatic filariasis; MDA, mass drug administration; Oncho, onchocerciasis

In total, only 4% of the SSA population living in areas at risk for onchocerciasis and/or LF live in high prevalence loiasis areas, although a further 5.7% live in areas of moderate transmission. Within high loiasis prevalence populations, we estimate between 197,000 and 1.2 million people to be at risk of ivermectin-associated SAEs, considering a minimum and maximum prevalence of very high intensity of loiasis infection (densities ≥ 30,000 mf/ml) of 2 and 12% respectively [26]). Further national estimates are provided in Table S2 of Additional file 1. It is noteworthy that a third of the areas potentially at high risk of SAEs are currently reported as being under MDA treatment for LF and/or onchocerciasis (Fig. 1), according to data available at the ESPEN portal [41]. This may have reduced the intensity of loiasis transmission in these areas and consequently, reduced the numbers at risk of SAEs in areas under treatment. There is evidence of persistent high transmission in loiasis endemic areas however, even after several years of intensive community-directed treatment with ivermectin (CDTi) [64].

**Fig. 2:**
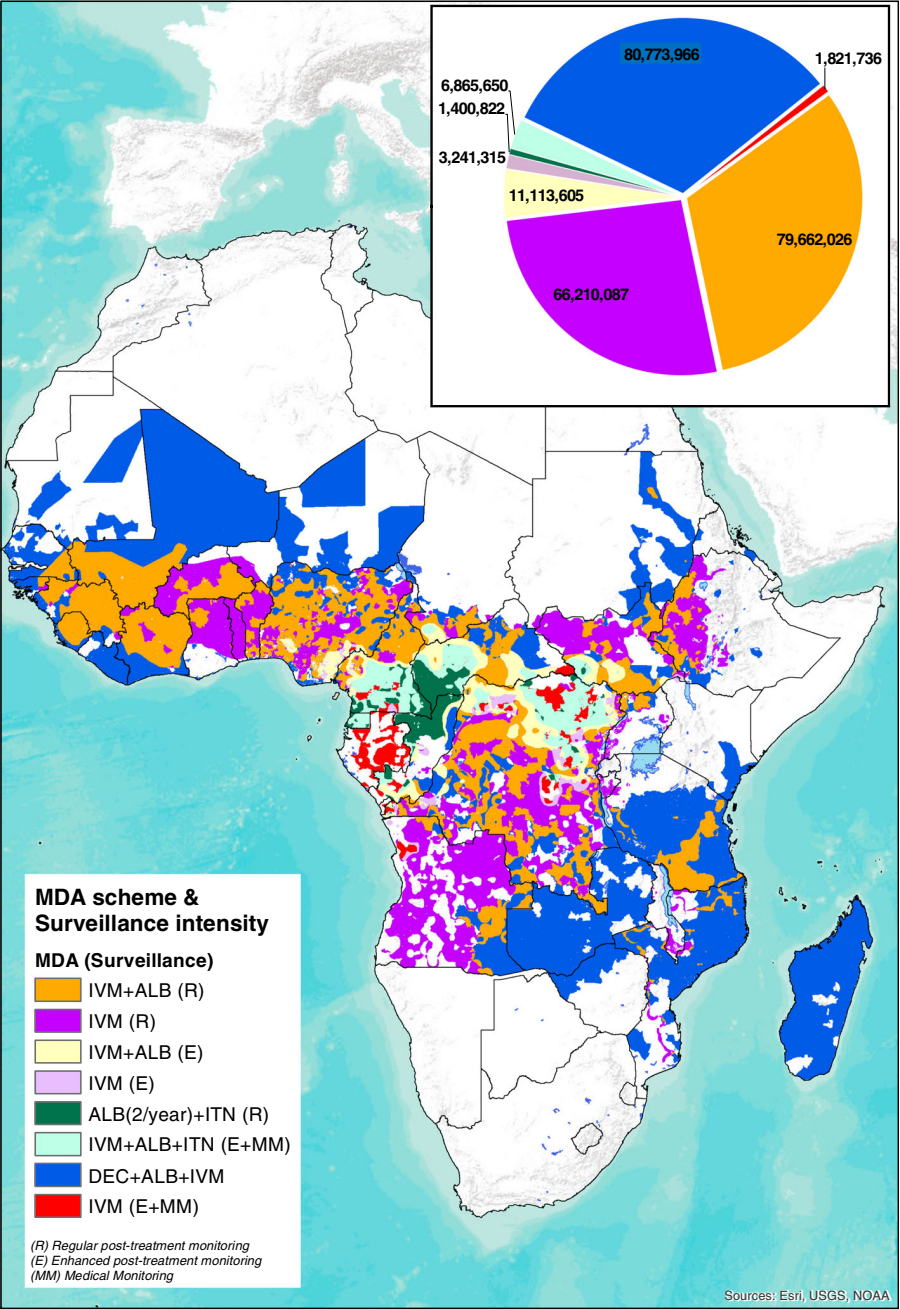
Suitable mass drug administration (MDA) and “Test & Treat” based schemes tailored to the type and level of co-endemicity of three major filarial infections in sub-Saharan Africa. The chart graph shows the overall population that may potentially benefit from different MDA schemes. *Abbreviations*: ALB, albendazole; DEC, diethylcarbamazine; E, enhanced post-treatment monitoring for rapid determination of potential loiasis-related SAEs; IVM, ivermectin; ITN, insecticide-treated nets; MDA, mass drug administration; MM, medical monitoring at the community during 3–4 days after MDA; R, regular monitoring of drug effects on treated communities; SAE, severe adverse event

Within these high loiasis areas, only a small proportion of the population requiring MDA would be suitable for targeting with twice-yearly albendazole (non-onchocerciasis endemic, LF endemic), confined primarily to Cameroon, Democratic Republic of Congo (DRC) and Central African Republic (CAR). The vast majority (close to 8.7 million people) are predicted to live in areas with onchocerciasis, where test-and-treat strategies are required (Fig. 2 and Table 2). Of these, 7 million also live in areas potentially co-endemic for LF (not accounting for possible over-diagnosis of LF in *L. loa* endemic areas).

**Table 2:** Estimates of populations living in areas endemic for three major filariases in Africa, which may be targeted with tailored mass drug administration (MDA) schemes according to the level of co-endemicity of the filarial nematode species

	Estimates of populations living in areas potentially targetable with different MDA schemes							
	IVM + ALB	IVM	IVM + ALB (E)^a^	IVM (E)^a^	ALB(2/year) + ITN^a^	IVM + ALB + ITN (E + MM)^a^	DEC + ALB + IVM^b^	IVM (E + MM)^a^
Eastern Africa	8,413,869	20,963,598	–	–	–	–	45,192,922	–
Burundi	–	1,390,645	–	–	–	–	–	–
Eritrea	–	–	–	–	–	–	104,459	–
Ethiopia	2,340,296	14,392,313	–	–	–	–	1,161,307	–
Kenya	–	–	–	–	–	–	1,045,022	–
Madagascar	–	–	–	–	–	–	13,557,237	–
Malawi	–	2,048,395	–	–	–	–	–	–
Mozambique	493,510	742,771	–	–	–	–	10,369,358	–
Rwanda	–	38,195	–	–	–	–	–	–
Uganda	1,426,558	2,174,834	–	–	–	–	992,624	–
Tanzania	4,153,505	176,445	–	–	–	–	10,262,625	–
Zambia	–	–	–	–	–	–	6,426,817	–
Zimbabwe	–	–	–	–	–	–	1,273,473	–
Middle Africa	17,259,697	18,580,670	7,040,636	2,498,132	1,381,558	6,636,062	4,154,028	1,741,016
Angola	225,104	5,670,871	–	111,473	323	2314	116,774	244,993
Cameroon	3,064,962	435,486	1,270,793	245,554	331,363	1,548,497	635,665	251,586
CAR	555,240	5358	681,391	3062	697,014	756,080	60,605	33,118
Chad	1,139,547	857,065	476,212	25,770	–	211,614	363,059	–
Congo	37,729	3026	101,911	5767	39,154	29,242	27,589	9924
DRC	12,237,115	11,608,864	4,483,037	2,104,428	222,533	3744,58	2,950,335	1,038,597
Equatorial Guinea	–	–	27,293	–	91,172	343,735	–	–
Gabon	–	–	–	2078	–	–	–	162,798
Northern Africa	3,045,672	3,046,221	509,869	18,858	19,263	208,310	4,688,564	8669
South Sudan	2,998,136	2,969,800	509,869	18,858	19,263	208,310	843,150	8669
Sudan	47,536	76,421	–	–	–	–	3,845,414	–
Western Africa	50,942,788	23,619,598	3,563,101	724,325	–	21,278	26,738,452	72,050
Benin	1,821,021	1,847,795	–	–	–	–	97,670	–
Burkina Faso	4,332,528	5,527,801	–	–	–	–	–	–
Ghana	307,396	2,030,355	–	–	–	–	84,886	–
Guinea	3,390,416	227,213	–	–	–	–	1,461,344	–
Guinea-Bissau	176,095	–	–	–	–	–	524,123	–
Liberia	42,021	–	–	–	–	–	1,964,905	–
Mali	5,959,767	6758	–	–	–	–	2,861,263	–
Mauritania	–	–	–	–	–	–	700,830	–
Niger	78,987	842,637	–	–	–	–	4,837,755	–
Nigeria	25,275,083	10,052,102	3,563,101	724,325	–	21,278	7,974,187	72,050
Senegal	412,921	–	–	–	–	–	2,883,203	–
Sierra Leone	2,688,260	–	–	–	–	–	683,589	–
Togo	–	2,670,471	–	–	–	–	–	–
Côte d’Ivoire	6,458,292	414,466	–	–	–	–	2,664,695	–
Grand total	79,662,026	66,210,087	11,113,605	3,241,315	1,400,822	6,865,650	80,773,966	1,821,736

^a^Test & Treat (measure *Loiasis* microfilaraemia load before treatment and exclude those with > 30,000 mf/ml)

^b^Triple therapy with DEC is not yet recommended in countries where onchocerciasis is endemic. Re-evaluation of current endemicity is now considered in areas that were classified as hypoendemic by REMO mapping

*Abbreviations*: *ALB* albendazole, *CAR* Central African Republic, *DEC* diethylcarbamazine, *DRC* Democratic Republic of Congo, *E* enhanced post-treatment monitoring for rapid determination of potential loiasis-related SAEs, *IVM* ivermectin, *ITN* insecticide-treated nets, *MDA* mass drug administration, *MM* medical monitoring at the community during 3–4 days after MDA; *SAE* severe adverse even

**Fig. 3:**
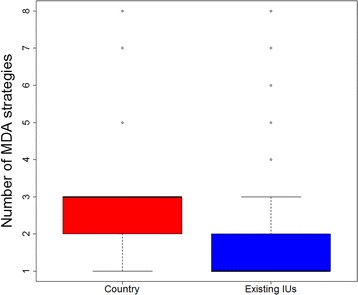
Variety of MDA schemes by country and implementation unit (IU) according to filariasis co-endemicity. The y-axis displays the number of IUs in which 1 to 8 different MDA schemes would be applicable according to the distribution and overlapping of loiasis, onchocerciasis and lymphatic filariasis

Considering the heterogeneity of disease distributions further, Fig. 3 highlights the median number of MDA strategies required within countries and existing IUs. Only six endemic countries require just one MDA strategy, with the majority requiring up to four. For some countries in Middle Africa (including CAR and DRC) up to eight different strategies are indicated. Even within implementation units, more than one MDA strategy is suggested for just over half (1775/3564) of all endemic units due to fine scale spatial heterogeneity of transmission, with 653 implementation units across SSA (primarily in Cameroon, DRC, Chad, Congo and Nigeria) potentially requiring between three and eight strategies. These patterns are emphasised Additional file 1: Figures S3 and S4, further demonstrating how patterns of co-endemicity vary within very small geographical areas, and how this information might guide the tailoring of local treatment strategies.

These maps are intended to initiate discussion around tailored treatment strategies, rather than to provide definitive recommendations. In particular, suggested recommendations for each IU presented here do not consider co-endemicity of other IUs, either in the same or in neighbouring countries. This is particularly important when considering whether an IDA-based MDA strategy is appropriate. It is notable that, when taking a stricter approach of only implementing triple drug therapy in countries non-endemic for onchocerciasis, the population that would benefit drops to 23,107,838 living across 6 countries.

Some important limitations should be acknowledged. First, the maps have been built upon predictive models using historic data. Each has an inherent degree of uncertainty, and should, wherever possible, be validated by national survey data. For example, nodule palpation for onchocerciasis can give false positive results in non-endemic areas [65] and lack sensitivity in areas of low sensitivity [66]. Nodule prevalence surveys were designed to delineate areas to be treated, namely, those with a nodule prevalence higher than 20% (indicative of at least mesoendemicity). Subsequently, for the purpose of elimination, it was agreed that the treatment boundaries need to be expanded to ensure that there remain no untreated onchocerciasis foci that might pose a future threat of reinfection. The nodule prevalence threshold below which we can assume there is no onchocerciasis transmission is still under discussion. Nevertheless, a 5% threshold has been suggested on the basis of non-onchocercal ‘nodule’ prevalence around 2% in endemic areas [67]. Such assumption will require further investigation.

Recent parasitological surveys have shown many areas considered as hypoendemic no longer to be endemic [68]. Furthermore, as noted above some areas highly endemic for loiasis in Middle Africa may require to be remapped for LF due to potential cross-reactivity of the ICT cards.

Secondly, the onchocerciasis and loiasis estimates reflect disease distribution prior to the scale up of mass treatment. Although efforts have been made to exclude areas considered as having interrupted transmission, we did not account for potential reduction on the intensity of loiasis transmission due to successive MDA rounds with ivermectin when estimating population potentially at-risk of suffering SAEs. Lastly, the incidence of loiasis-associated SAEs following ivermectin administration is seen to vary substantially within co-endemic areas [69], which points to the existence of other as yet unidentified risk factors that require further exploration.

## Conclusions

Substantial advances have been made towards the elimination of onchocerciasis and LF in SSA [5, 6]. Despite prolonged control activities however, many endemic areas are still experiencing ongoing transmission. Taken together with the risk of loiasis-related SAEs, issues of efficacy and appropriateness for existing treatment strategies remains of major concern. The work presented here highlights settings suitable for innovative MDA regimens and integrated control, which may help to address these concerns. Further work is required to test new strategies in programmatic settings, providing the empirical evidence needed to guide efforts towards the 2020 goals and beyond.

## Additional file

Additional file 1: Pan-African and country-specific (i.e. Cameroon and Democratic Republic of Congo) maps for lymphatic filariasis (LF); LF and onchocerciasis co-endemicity; LF, onchocerciasis and loiasis co-endemicity, and population estimates aggregated by African region. **Text S1.** Pan-African maps of lymphatic filariasis (LF). **Figure S1.** Pan-African map of LF endemicity at the level of implementation units (IU). **Figure S2**. Pan-African map of co-endemic areas for LF and onchocerciasis. **Figure S3.** Map of loiasis, LF and onchocerciasis co-endemicity in eastern Cameroon. **Figure S4.** Map of loiasis, LF and onchocerciasis co-endemicity in Oriental province, DRC. **Table S1.** Estimates of population living in endemic areas for lymphatic filariasis (LF) and/or onchocerciasis (oncho). **Table S2.** Population estimates for co-endemic areas of lymphatic filariasis, onchocerciasis and high prevalence of loiasis. (DOCX 2183 kb)

## Abbreviations

APOC: African Programme for Onchocerciasis Control
CAR: Central African Republic
CDTi: Community directed treatment with ivermectin
CFA: Circulating filarial antigen
DEC: Diethylcarbamazine citrate
DRC: Democratic Republic of Congo
ESPEN: Expanded special programme for elimination of neglected tropical diseases
EWH: Eye worm history
GIS: Geographical information system
ICT: Immunochromatographic card test
IDA: Ivermectin-diethylcarbamazine-albendazole triple therapy
IU: Implementation unit
LF: Lymphatic filariasis
MDA: Mass drug administration
MEC: Mectizan expert committee
mf: microfilariae
NTD: Neglected tropical disease
OCP: Onchocerciasis Control Programme in West Africa
PCT: Preventive chemotherapy and transmission control
RAPLOA: Rapid assessment procedure for *Loa loa*
SAE: Severe adverse event
SSA: Sub-Saharan Africa
TCC: Technical consultative committee
WHO: World Health Organization

## References

[1] Taylor MJ, Hoerauf A, Bockarie M (2010). Lymphatic filariasis and onchocerciasis. Lancet.

[2] Boussinesq M (2006). Loiasis. Ann Trop Med Parasitol.

[3] Chesnais CB, Takougang I, Paguele M, Pion SD, Boussinesq M (2017). Excess mortality associated with loiasis: a retrospective population-based cohort study. Lancet Infect Dis.

[4] WHO (2012). Accelerating work to overcome the global impact of neglected tropical diseases - a roadmap for implementation.

[5] Crump A, Morel CM, Omura S (2012). The onchocerciasis chronicle: from the beginning to the end?. Trends Parasitol.

[6] Ottesen EA, Hooper PJ, Bradley M, Biswas G (2008). The global programme to eliminate lymphatic filariasis: health impact after 8 years. PLoS Negl Trop Dis.

[7] Ichimori K, King JD, Engels D, Yajima A, Mikhailov A, Lammie P, et al. Global Programme to Eliminate Lymphatic Filariasis: the processes underlying programme success. PLoS Negl Trop Dis. 2014;8(12):e3328. 10.1371/journal.pntd.0003328 PMC4263400 25502758

[8] Sturchio JL (2001). The case of ivermectin: lessons and implications for improving access to care and treatment in developing countries. Community Eye Health.

[9] Rebollo MP, Bockarie MJ (2013). Toward the elimination of lymphatic filariasis by 2020: treatment update and impact assessment for the endgame. Expert Rev Anti-Infect Ther.

[10] Basáñez MG, Pion SDS, Boakes E, Filipe JAN, Churcher TS, Boussinesq M (2008). Effect of single-dose ivermectin on *Onchocerca volvulus*: a systematic review and meta-analysis. Lancet Infect Dis.

[11] de Kraker ME, Stolk WA, van Oortmarssen GJ, Habbema JD (2006). Model-based analysis of trial data: microfilaria and worm-productivity loss after diethylcarbamazine-albendazole or ivermectin-albendazole combination therapy against *Wuchereria bancrofti*. Tropical Med Int Health.

[12] Dyson L, Stolk WA, Farrell SH, Hollingsworth TD (2017). Measuring and modelling the effects of systematic non-adherence to mass drug administration. Epidemics.

[13] Stolk WA, Stone C, de Vlas SJ (2015). Modelling lymphatic filariasis transmission and control: modelling frameworks, lessons learned and future directions. Adv Parasitol.

[14] Plaisier AP, van Oortmarssen GJ, Remme J, Habbema JD (1991). The reproductive lifespan of *Onchocerca volvulus* in west African savanna. Acta Trop.

[15] Kuesel AC (2016). Research for new drugs for elimination of onchocerciasis in Africa. Int J Parasitol Drugs Drug Resist.

[16] Thomsen EK, Sanuku N, Baea M, Satofan S, Maki E, Lombore B (2016). Efficacy, safety, and pharmacokinetics of coadministered diethylcarbamazine, albendazole, and ivermectin for treatment of Bancroftian filariasis. Clin Infect Dis.

[17] Irvine MA, Stolk WA, Smith ME, Subramanian S, Singh BK, Weil GJ (2017). Effectiveness of a triple-drug regimen for global elimination of lymphatic filariasis: a modelling study. Lancet Infect Dis.

[18] Molyneux DH, Hopkins A, Bradley MH, Kelly-Hope LA (2014). Multidimensional complexities of filariasis control in an era of large-scale mass drug administration programmes: a can of worms. Parasit Vectors.

[19] Lariviere M, Vingtain P, Aziz M, Beauvais B, Weimann D, Derouin F (1985). Double-blind study of ivermectin and diethylcarbamazine in African onchocerciasis patients with ocular involvement. Lancet.

[20] Gardon J, Gardon-Wendel N (1997). Demanga Ngangue, Kamgno J, Chippaux JP, Boussinesq M. Serious reactions after mass treatment of onchocerciasis with ivermectin in an area endemic for *Loa loa* infection. Lancet.

[21] Herrick JA, Legrand F, Gounoue R, Nchinda G, Montavon C, Bopda J (2017). Posttreatment reactions after single-dose diethylcarbamazine or ivermectin in subjects with *Loa loa* infection. Clin Infect Dis.

[22] Thomson MC, Obsomer V, Dunne M, Connor SJ, Molyneux DH (2000). Satellite mapping of *Loa loa* prevalence in relation to ivermectin use in west and central Africa. Lancet.

[23] WHO (2012). Provisional strategy for interrupting LF transmission in loiasis-endemic countries.

[24] Awadzi K, Edwards G, Duke BOL, Opoku NO, Attah SK, Addy ET (2003). The co-administration of ivermectin and albendazole - safety, pharmacokinetics and efficacy against *Onchocerca volvulus*. Ann Trop Med Parasitol.

[25] Mectizan Expert Committee/APOC Technical Consultative Committee (2004). Guidelines for use of Mectizan in areas co-endemic for onchocerciasis and loiasis.

[26] Addiss DG, Rheingans R, Twum-Danso NA, Richards FO (2003). A framework for decision-making for mass distribution of Mectizan^(R)^ in areas endemic for *Loa loa*. Filaria J.

[27] D’Ambrosio MV, Bakalar M, Bennuru S, Reber C, Skandarajah A, Nilsson L (2015). Point-of-care quantification of blood-borne filarial parasites with a mobile phone microscope. Sci Transl Med.

[28] Drame PM, Fink DL, Kamgno J, Herrick JA, Nutman TB (2014). Loop-mediated isothermal amplification for rapid and semiquantitative detection of *Loa loa* infection. J Clin Microbiol.

[29] Geary TG (2016). A step toward eradication of human filariases in areas where *Loa* is endemic. MBio.

[30] Taylor MJ, Hoerauf A, Townson S, Slatko BE, Ward SA (2014). Anti-*Wolbachia* drug discovery and development: safe macrofilaricides for onchocerciasis and lymphatic filariasis. Parasitology.

[31] Grobusch MP, Kombila M, Autenrieth I, Mehlhorn H, Kremsner PG (2003). No evidence of *Wolbachia* endosymbiosis with *Loa loa* and *Mansonella perstans*. Parasitol Res.

[32] Walker M, Specht S, Churcher TS, Hoerauf A, Taylor MJ, Basáñez MG (2015). Therapeutic efficacy and macrofilaricidal activity of doxycycline for the treatment of river blindness. Clin Infect Dis.

[33] Brito M, Paulo R, Van-Dunem P, Martins A, Unnasch TR, Novak RJ (2017). Rapid integrated clinical survey to determine prevalence and co-distribution patterns of lymphatic filariasis and onchocerciasis in a *Loa loa* co-endemic area: the Angolan experience. Parasite Epidemiol Control.

[34] Zouré HG, Noma M, Tekle AH, Amazigo UV, Diggle PJ, Giorgi E, et al. The geographic distribution of onchocerciasis in the 20 participating countries of the African Programme for Onchocerciasis Control: (2) pre-control endemicity levels and estimated number infected. Parasit Vectors. 2014;7:326. 10.1186/1756-3305-7-326 PMC4222889 25053392

[35] Moraga P, Cano J, Baggaley R, Gyapong J, Njenga S, Nikolay B (2015). Modelling the distribution and transmission intensity of lymphatic filariasis in sub-Saharan Africa prior to scaling up interventions: integrated use of geostatistical and mathematical modelling. Parasit Vectors.

[36] Zouré HG, Wanji S, Noma M, Amazigo UV, Diggle PJ, Tekle AH, et al. The geographic distribution of *Loa loa* in Africa: results of large-scale implementation of the Rapid Assessment Procedure for Loiasis (RAPLOA). PLoS Negl Trop Dis. 2011;5(6):e1210. 10.1371/journal.pntd.0001210 PMC3125145 21738809

[37] Kelly-Hope LA, Cano J, Stanton MC, Bockarie MJ, Molyneux DH (2014). Innovative tools for assessing risks for severe adverse events in areas of overlapping *Loa loa* and other filarial distributions: the application of micro-stratification mapping. Parasit Vectors.

[38] Okorie PN, Ademowo GO, Saka Y, Davies E, Okoronkwo C, Bockarie MJ (2013). Lymphatic filariasis in Nigeria; micro-stratification overlap mapping (MOM) as a prerequisite for cost-effective resource utilization in control and surveillance. PLoS Negl Trop Dis.

[39] O'Hanlon SJ, Slater HC, Cheke RA, Boatin BA, Coffeng LE, Pion SD (2016). Model-based geostatistical mapping of the prevalence of *Onchocerca volvulus* in West Africa. PLoS Negl Trop Dis.

[40] Flueckiger RM, Nikolay B, Gelderblom HC, Smith JL, Haddad D, Tack W (2015). Integrating data and resources on neglected tropical diseases for better planning: the NTD mapping tool (NTDmap.org). PLoS Negl Trop Dis.

[41] Expanded Special Project for Elimination of Neglected Tropical Diseases (ESPEN): NTD portal. Available: http://ntd.afro.who.int/en/espen/home. Accessed 13 Jan 2018.

[42] World Health Organization. Neglected tropical diseases. PCT Databank - Country Profiles 2010. Available: http://www.who.int/neglected_diseases/preventive_chemotherapy/databank/CP_CountryName.pdf. Accessed 13 Jan 2018.

[43] WHO (2000). Operational guidelines for rapid mapping of bancroftian filariasis in Africa (WHO/CDS/CPE/CEE/2000.9).

[44] African Programme for Onchocerciasis Control. Guidelines for revising ivermectin treatment boundaries within the context of onchocerciasis elimination. WHO/MG/15.21. Geneva: World Health Organization; 2015. Available: http://www.who.int/apoc/ATS_Report_Annex1_APOC_Guidelines_for_revising_IVM_Tx_boundaries.pdf. Accessed 13 Jan 2018.

[45] African Programme for Onchocerciasis Control (2010). Conceptual and operational framework of Onchocerciasis elimination with Ivermectin treatment.

[46] Traore MO, Sarr MD, Badji A, Bissan Y, Diawara L, Doumbia K (2012). Proof-of-principle of onchocerciasis elimination with ivermectin treatment in endemic foci in Africa: final results of a study in Mali and Senegal. PLoS Negl Trop Dis.

[47] Diawara L, Traore MO, Badji A, Bissan Y, Doumbia K, Goita SF (2009). Feasibility of onchocerciasis elimination with ivermectin treatment in endemic foci in Africa: first evidence from studies in Mali and Senegal. PLoS Negl Trop Dis.

[48] Tekle AH, Elhassan E, Isiyaku S, Amazigo UV, Bush S, Noma M, et al. Impact of long-term treatment of onchocerciasis with ivermectin in Kaduna state, Nigeria: first evidence of the potential for elimination in the operational area of the African Programme for Onchocerciasis Control. Parasit Vectors. 2012;5:28. 10.1186/1756-3305-5-28 PMC3296569 22313631

[49] Zarroug IM, Hashim K, ElMubark WA, Shumo ZA, Salih KA, ElNojomi NA (2016). The first confirmed elimination of an onchocerciasis focus in Africa: Abu Hamed, Sudan. Am J Trop Med Hyg.

[50] Lakwo T, Garms R, Wamani J, Tukahebwa EM, Byamukama E, Onapa AW (2017). Interruption of the transmission of *Onchocerca volvulus* in the Kashoya-Kitomi focus, western Uganda by long-term ivermectin treatment and elimination of the vector *Simulium neavei* by larviciding. Acta Trop.

[51] Lakwo TL, Garms R, Rubaale T, Katabarwa M, Walsh F, Habomugisha P (2013). The disappearance of onchocerciasis from the Itwara focus, western Uganda after elimination of the vector *Simulium neavei* and 19 years of annual ivermectin treatments. Acta Trop.

[52] Katabarwa MN, Eyamba A, Nwane P, Enyong P, Yaya S, Baldiagaï J, et al. Seventeen years of annual distribution of ivermectin has not interrupted onchocerciasis transmission in North Region, Cameroon. Am J Trop Med Hyg. 2011;85(6):1041–9. 10.4269/ajtmh.2011.11-0333 PMC3225149 22144441

[53] Katabarwa MN, Lakwo T, Habomugisha P, Agunyo S, Byamukama E, Oguttu D (2013). Transmission of *Onchocerca volvulus* continues in Nyagak-Bondo focus of northwestern Uganda after 18 years of a single dose of annual treatment with ivermectin. Am J Trop Med Hyg..

[54] Eisenbarth A, Achukwi MD, Renz A (2016). Ongoing transmission of *Onchocerca volvulus* after 25 years of annual ivermectin mass treatments in the Vina du Nord river valley, in North Cameroon. PLoS Negl Trop Dis.

[55] Walker M, Stolk WA, Dixon MA, Bottomley C, Diawara L, Traore MO (2017). Modelling the elimination of river blindness using long-term epidemiological and programmatic data from Mali and Senegal. Epidemics.

[56] Wilson NO, Badara Ly A, Cama VA, Cantey PT, Cohn D, Diawara L (2016). Evaluation of lymphatic filariasis and onchocerciasis in three Senegalese districts treated for onchocerciasis with ivermectin. PLoS Negl Trop Dis.

[57] Linard C, Gilbert M, Snow RW, Noor AM, Tatem AJ (2012). Population distribution, settlement patterns and accessibility across Africa in 2010. PLoS One.

[58] Speich B, Ali SM, Ame SM, Bogoch II, Alles R, Huwyler J (2015). Efficacy and safety of albendazole plus ivermectin, albendazole plus mebendazole, albendazole plus oxantel pamoate, and mebendazole alone against *Trichuris trichiura* and concomitant soil-transmitted helminth infections: a four-arm, randomised controlled trial. Lancet Infect Dis.

[59] Gonzalez P, Gonzalez FA, Ueno K (2012). Ivermectin in human medicine, an overview of the current status of its clinical applications. Curr Pharm Biotechnol.

[60] World Health Organization. Neglected tropical diseases. Preventive Chemotherapy databank. Available: http://www.who.int/neglected_diseases/preventive_chemotherapy/databank/en/index.htm. Accessed 13 Jan 2018.

[61] Wanji S, Amvongo-Adjia N, Koudou B, Njouendou AJ, Chounna Ndongmo PW, Kengne-Ouafo JA, et al. Cross-reactivity of filariais ICT cards in areas of contrasting endemicity of *Loa loa* and *Mansonella perstans* in Cameroon: implications for shrinking of the lymphatic filariasis map in the Central African region. PLoS Negl Trop Dis. 2015;9(11):e0004184. 10.1371/journal.pntd.0004184 PMC4636288 26544042

[62] Pion SDS, Montavon C, Chesnais CB, Kamgno J, Wanji S, Klion AD (2016). Positivity of antigen tests used for diagnosis of lymphatic filariasis in individuals without *Wuchereria bancrofti* infection but with high *Loa loa* microfilaremia. Am J Trop Med Hyg.

[63] Wanji S, Amvongo-Adjia N, Njouendou AJ, Kengne-Ouafo JA, Ndongmo WP, Fombad FF (2016). Further evidence of the cross-reactivity of the Binax NOW(R) Filariasis ICT cards to non-*Wuchereria bancrofti* filariae: experimental studies with *Loa loa* and *Onchocerca ochengi*. Parasit Vectors.

[64] Kouam MK, Tchatchueng-Mbougua JB, Demanou M, Boussinesq M, Pion SDS, Kamgno J. Impact of repeated ivermectin treatments against onchocerciasis on the transmission of loiasis: an entomologic evaluation in central Cameroon. Parasit Vectors 2013;6(1):283. 10.1186/1756-3305-6-283 PMC3849770 24289520

[65] Kim YE, Remme JHF, Steinmann P, Stolk WA, Roungou J-B, Tediosi F (2015). Control, elimination, and eradication of river blindness: scenarios, timelines, and ivermectin treatment needs in Africa. PLoS Negl Trop Dis.

[66] Duerr HP, Raddatz G, Eichner M (2008). Diagnostic value of nodule palpation in onchocerciasis. Trans R Soc Trop Med Hyg.

[67] Fischer P, Kipp W, Bamuhiga J, Binta-Kahwa J, Kiefer A, Büttner DW (1993). Parasitological and clinical characterization of *Simulium neavei*-transmitted onchocerciasis in western Uganda. Trop Med Parasitol.

[68] African Programme for Onchocerciasis Control. The WHO African Programme for Onchocerciasis Control Progress Report 2013 (1st September 2012 - 31st August 2013). JAF19.5. World Health Organization/APOC; 2013. Available: http://www.who.int/apoc/publications/JAF195_EN_APOC_PR2013_OK.pdf?ua=1. Accessed 13 Jan 2018.

[69] Twum-Danso NA, Meredith SE (2003). Variation in incidence of serious adverse events after onchocerciasis treatment with ivermectin in areas of Cameroon co-endemic for loiasis. Tropical Med Int Health.

