# Supplementary material for: Identifying co-endemic areas for major filarial infections in sub-Saharan Africa: seeking synergies and preventing severe adverse events during mass drug administration campaigns
Source: Parasit Vectors. 2018 Jan 31;11:70. doi: 10.1186/s13071-018-2655-5 (PMC5791223; doi:10.1186/s13071-018-2655-5)
Supplement: Additional file 1: — Pan-African and country-specific (i.e. Cameroon and Democratic Republic of Congo) maps for lymphatic filariasis (LF); LF and onchocerciasis co-endemicity; LF, onchocerciasis and loiasis co-endemicity, and population estimates aggregated by African region. Text S1. Pan-African maps of lymphatic filariasis (LF). Figure S1. Pan-African map of LF endemicity at the level of implementation units (IU). Figure S2. Pan-African map of co-endemic areas for LF and onchocerciasis. Figure S3. Map of loiasis, LF and onchocerciasis co-endemicity in eastern Cameroon. Figure S4. Map of loiasis, LF and onchocerciasis co-endemicity in Oriental province, DRC. Table S1. Estimates of population living in endemic areas for lymphatic filariasis (LF) and/or onchocerciasis (oncho). Table S2. Population estimates for co-endemic areas of lymphatic filariasis, onchocerciasis and high prevalence of loiasis. (DOCX 2183 kb) [file 13071_2018_2655_MOESM1_ESM.docx]

**Additional file 1**

**Pan-African and country-specific (i.e. Cameroon and Democratic Republic of Congo) maps for lymphatic filariasis (LF); LF and onchocerciasis co-endemicity; LF, onchocerciasis and loiasis co-endemicity, and population estimates aggregated by African region**

**Text S1. *Pan-African maps of lymphatic filariasis (LF)***

A map of lymphatic filariasis (LF) endemicity at implementation unit (IU) level was produced from the most updated information available (Figure S1A) using information from the neglected tropical disease (NTD) portal recently launched by the Expanded Special Project for Elimination of Neglected Tropical Diseases (ESPEN)^[[1]](#footnote-1)^. This portal has been conceived as a repository of mapping and surveillance data for five major NTDs tackled by the preventive chemotherapy (PC-NTDs) strategy of the World Health Organization, namely, LF, onchocerciasis, soil-transmitted helminthiases, schistosomiasis and trachoma. For eight of the countries^[[2]](#footnote-2)^ whose endemicity maps are not yet available at the ESPEN NTD portal, endemicity status of IU was obtained from the Preventive Chemotherapy and Transmission Control (PCT) databank [[1](#_ENREF_1)], which provides detailed epidemiological information for all NTDs by 2010. Previously endemic countries that have recently passed their Transmission Assessment Surveys (TAS, designed for making decisions about stopping LF treatment) and indeed have stopped treatment (and initiated post-treatment surveillance), were re-categorized as currently non-endemic (i.e. Malawi, Togo and The Gambia) [[2](#_ENREF_2), [3](#_ENREF_3)].

However, because for some countries nationwide mapping is either not yet completed or not publicly available at the aforementioned sources (i.e. Angola, Central African Republic, Republic of Congo, Sudan, South Sudan and Zimbabwe), we used a map of predicted *Wuchereria bancrofti* antigenaemia prevalence (based on the immunochromatographic card test (ICT) across sub-Saharan Africa recently published to complete the gaps in endemicity status [[4](#_ENREF_4)]. Besides this risk map, we also computed and modelled the probability that the prevalence of antigenaemia was equal to, or greater than the endemicity threshold of 1% prevalence (Figure S1B). In Figure S1B, areas situated within the deepest red colour (indicating probabilities of at least 75%) are those where there is a high probability that the infection threshold of at least 1%, circulating filarial antigen that is used to trigger mass drug administration (MDA) [[5](#_ENREF_5)], is exceeded. The lighter red areas (indicating probabilities of ≤ 25%) are those where there is a low probability of exceeding the infection threshold for MDA, and the intermediate colour ranges (>25% and ≤75%) can be considered as areas of uncertainty. We used the highest probability range to estimate the endemicity status of those IUs for which mapping data were not available at the present time (Figure S1C). Finally, urban extents (UEs), defined as areas with population densities ≥1,000 persons/km^2^, and peri-urban areas (>250 persons/km^2^ within a 15 km distance from UE edge) [[6](#_ENREF_6)], were masked out because they would be considered at low risk for LF transmission.

**Figure S1. Pan-African map of LF endemicity at the level of implementation units (IU)**


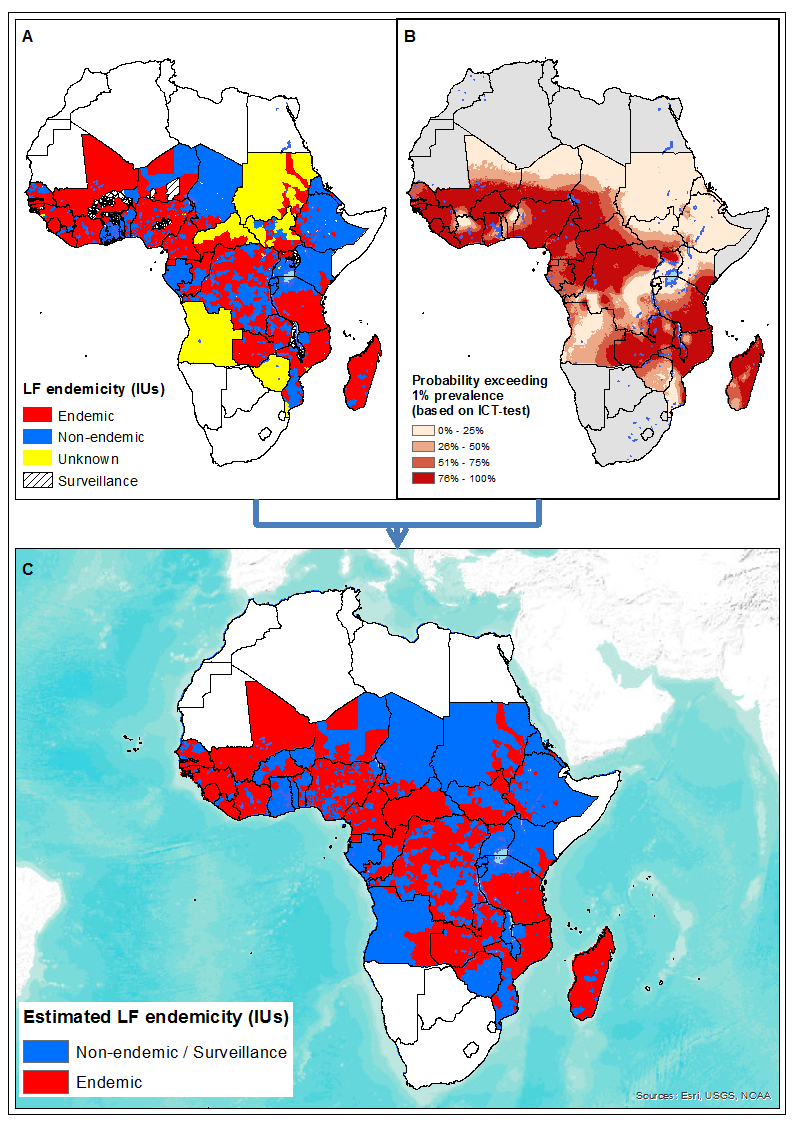
**Figure S2. Pan-African map of co-endemic areas for LF and onchocerciasis**

**
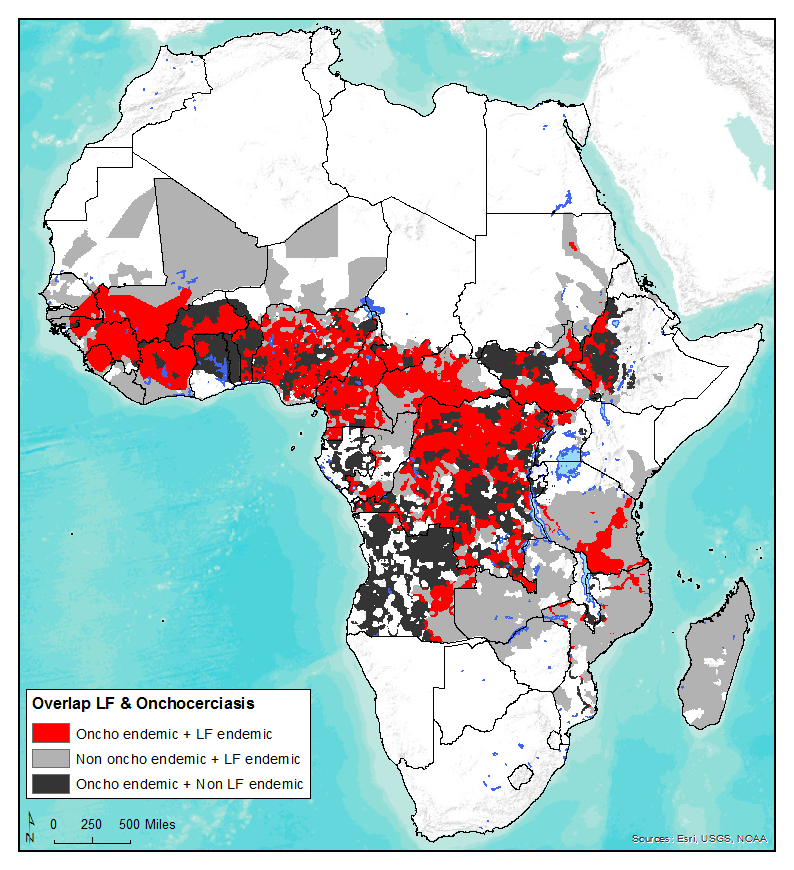
**

Dark red areas correspond to onchocerciasis (oncho) endemic areas that may benefit from mass drug administration (MDA) interventions based on ivermectin plus albendazole against lymphatic filariasis (LF). Dark grey areas correspond to non-endemic areas for LF but endemic for onchocerciasis and which require MDA or tailored MDA/alternative treatment strategies (see Table 1 of main text).

**Figure S3. Map of loiasis, LF and onchocerciasis co-endemicity in eastern Cameroon.** Suitable MDA strategies, according to the co-endemicity level of the three filarial infections, are highlighted for a subset of villages (green triangles and tabulated results). EWH = Prevalence of eye worm history.


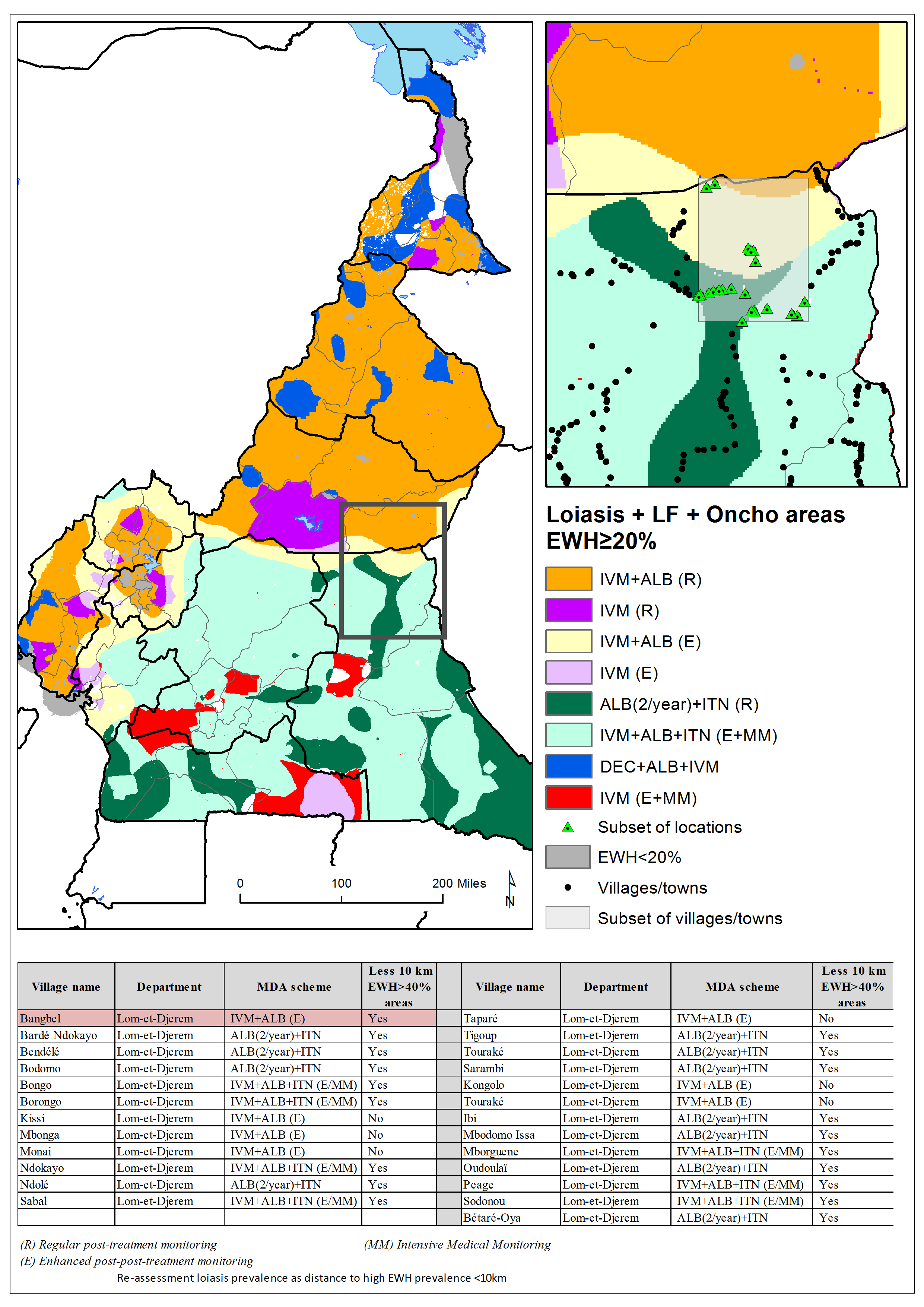


**Figure S4. Map of loiasis, LF and onchocerciasis co-endemicity in Oriental province, DRC.** Suitable MDA strategies, according to the co-endemicity level of the three filarial infections, are highlighted for a subset of villages (green triangles and tabulated results). EWH = Prevalence of eye worm history.

**
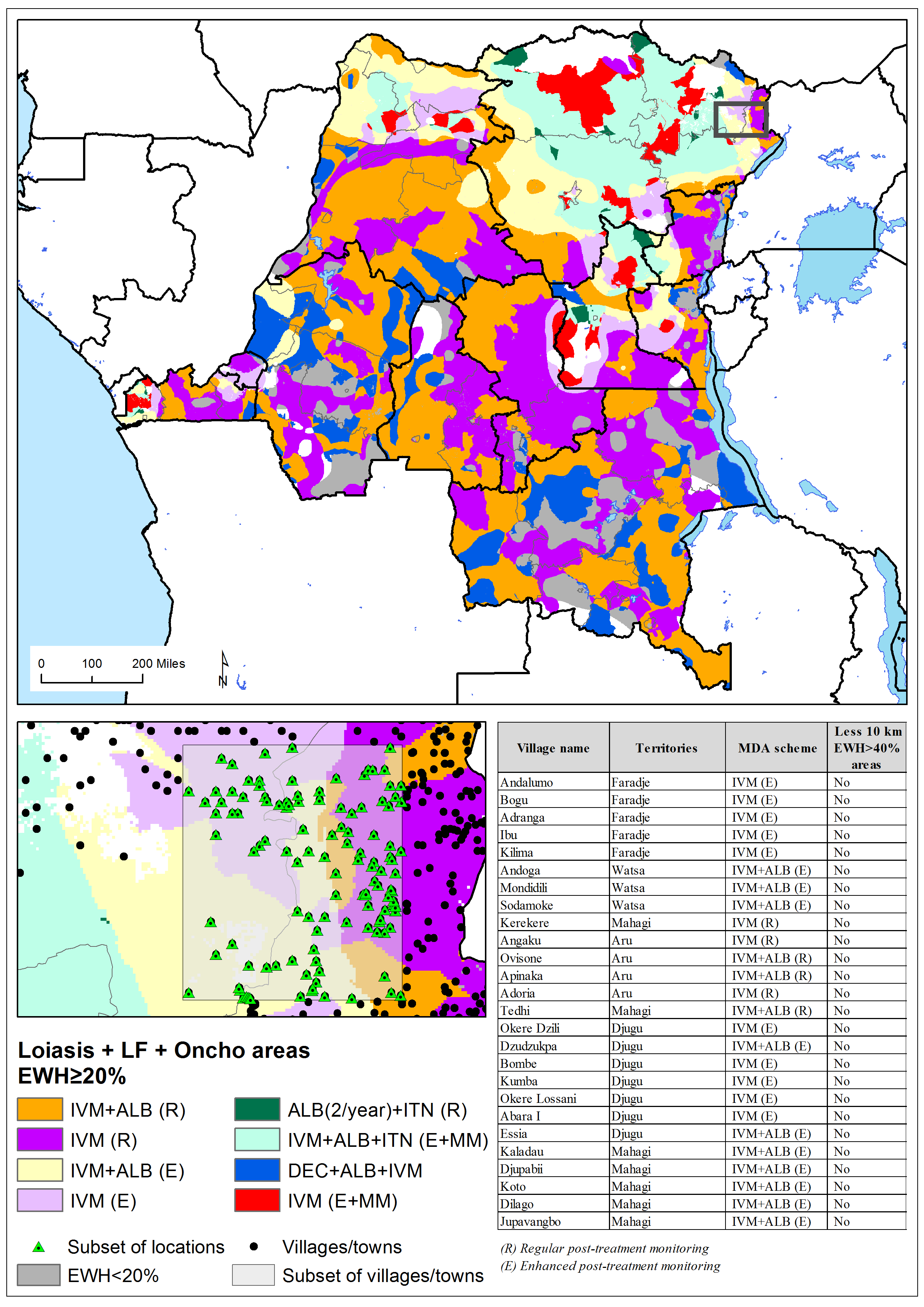
**

**Table S1 Estimates of population living in endemic areas for lymphatic filariasis (LF) and/or onchocerciasis (oncho).** Population estimates have been obtained at the implementation unit (IU) level for LF (i.e. mostly at health or administrative district level). CAR = Central African Republic.

**Table S2.** **Population estimates for co-endemic areas of lymphatic filariasis (LF), onchocerciasis and high prevalence of loiasis.** Estimates of individuals harbouring high *L. loa* microfilaraemia (≥30,000 mf/ml) were obtained considering the lowest and highest prevalence observed in areas where prevalence of EWH was greater than 20% [[7](#_ENREF_7)]. *Estimated from population density and prevalence in EWH≥20% areas. LB & UB = Lower & Upper bounds, respectively.

.

**Supplementary References**

1. World Health Organization G: PCT databank. Country profiles for countries in the WHO African Region (2010). <http://www.who.int/neglected_diseases/preventive_chemotherapy/databank/databank_CP_AFRO/en/> (2010).

2. Rebollo MP, Sambou SM, Thomas B, Biritwum NK, Jaye MC, Kelly-Hope L, et al. Elimination of lymphatic filariasis in the Gambia. PLoS Negl Trop Dis. 2015;9 3:e0003642.

3. WHO. Global programme to eliminate lymphatic filariasis: progress report, 2014. Releve epidemiologique hebdomadaire / Section d'hygiene du Secretariat de la Societe des Nations = Weekly epidemiological record / Health Section of the Secretariat of the League of Nations. 2015;90 38:489-504.

4. Moraga P, Cano J, Baggaley RF, Gyapong JO, Njenga SM, Nikolay B, et al. Modelling the distribution and transmission intensity of lymphatic filariasis in sub-Saharan Africa prior to scaling up interventions: integrated use of geostatistical and mathematical modelling. Parasites & vectors. 2015;8 1:560.

5. WHO. Monitoring and Epidemiological Assessment of Mass Drug Administration for the Global Programme to Eliminate Lymphatic Filariasis (GPELF). A Manual for National Elimination Programmes. World Health Organization, Geneva. 2011;WHO/HTM/NTD/PCT/2011.4.

6. Pullan RL, Brooker SJ. The global limits and population at risk of soil-transmitted helminth infections in 2010. Parasites & vectors. 2012;5:81.

7. Addiss DG, Rheingans R, Twum-Danso NA, Richards FO. A Framework for Decision-Making for Mass Distribution of Mectizan(R) in Areas Endemic for Loa loa. Filaria J. 2003;2 Suppl 1:S9.

1. ESPEN – NTD portal (http://ntd.afro.who.int/en/espen/home). [↑](#footnote-ref-1)
2. Benin, Equatorial Guinea, Liberia, Mozambique, Mauritius, Mayotte, Rwanda & The Gambia. [↑](#footnote-ref-2)
